# Transcatheter Coil Embolization of a Coronary Artery-Left Ventricular Fistula Associated with Single Coronary Artery Anomaly

**DOI:** 10.1155/2014/865490

**Published:** 2014-03-10

**Authors:** Ozlem Ozcan Celebi, Alper Canbay, Erdem Diker, Barbaros Çil, Kudret Aytemir, Ali Oto

**Affiliations:** ^1^Department of Cardiology, Medicana International Ankara Hastanesi, Söğütözü, Ankara, Turkey; ^2^Department of Radiology, Hacettepe University Medical School, Ankara, Turkey; ^3^Department of Cardiology, Hacettepe University Medical School, Ankara, Turkey

## Abstract

Single coronary artery anomaly associated with coronary fistula is a rare entity. Transcatheter coil embolization is the treatment of choice for coronary artery fistulas. In this case report, we describe a patient with both single coronary artery anomaly and coronary fistula who was successfully treated with coil embolization.

## 1. Introduction

Congenital coronary artery fistula is defined as a rare anomalous connection between a coronary artery and a major vessel or a cardiac chamber. It is a rare condition and is frequently diagnosed incidentally. Treatment of a congenital coronary artery fistula is a challenge for cardiologist.

## 2. Case Report

**Figure 1:**
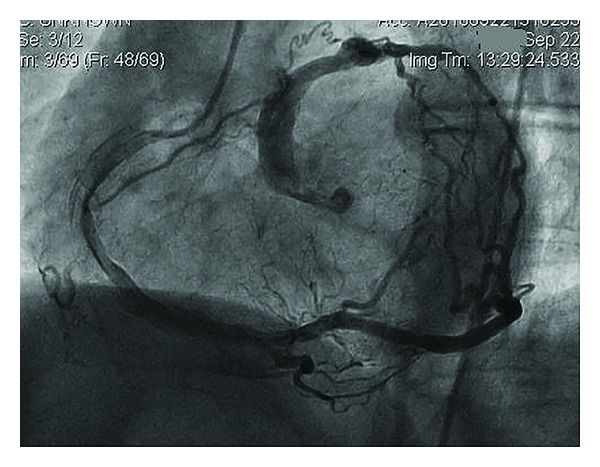
Coronary cine angiogram. Left anterior oblique projection shows a single large right coronary artery originated from the right aortic sinus and extended to the left and terminating with a tortuous fistulisation into a cardiac chamber.

**Figure 2:**
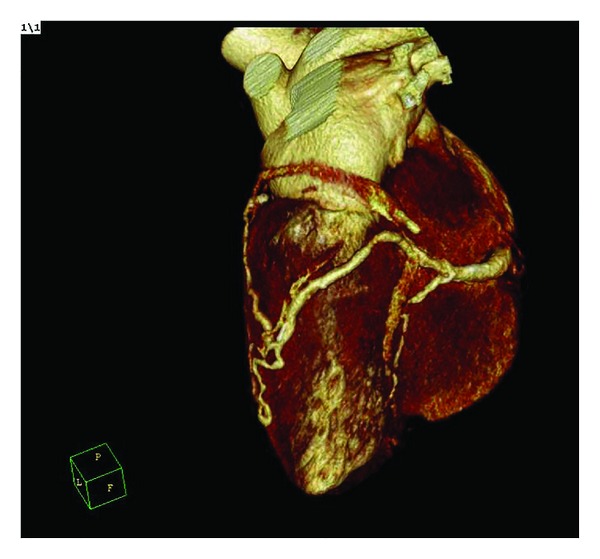
Multislice computed tomography. Volume rendering image shows that right coronary artery preceded in its regular pathway and gave branches to the left coronary system and then terminated with a fistula into the left ventricle.

**Figure 3:**
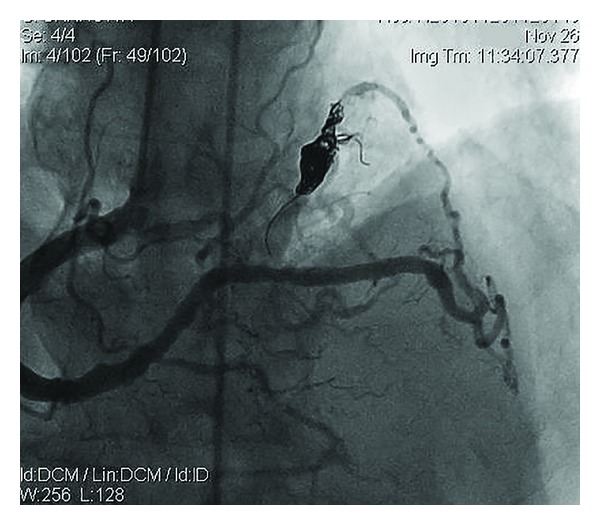
Coronary cine angiogram. Left anterior oblique projection shows single large right coronary artery after the coil embolization.

A 56-year-old male was admitted to our institution with anginal chest pain lasting for 3 months. He had no history of cardiac disease and no cardiac risk factors. Examination of cardiovascular system revealed a continuous murmur in the left parasternal border. The remainder of the examination was unremarkable. 12-lead surface electrocardiogram was normal. Transthoracic echocardiography revealed hypokinesia of inferior wall and mild mitral regurgitation. Left ventricular ejection fraction calculated by modified Simpson's method was 52%. In the light of these findings, selective coronary angiography was performed. A single coronary artery was arising from the right sinus of valsalva and terminating with fistulisation into a cardiac chamber (Figure 1). Anomalous coronary artery was dilated and no significant stenosis was encountered in any of the coronary arteries imaged. Multislice computed tomography showed origins of all coronary arteries arising from the right sinus of valsalva with a single ostium (Figure 2). The course of the anomalous coronary artery was not between the pulmonary and aortic trunci. Percutaneously, catheter-based coil embolization was performed to occlude the fistula. A standard 8-F guiding catheter was inserted into the right sinus of valsalva. A floppy guidewire was placed into the fistula and a microcatheter was advanced over the guidewire. The guidewire was withdrawn and a detachable coil was placed into the proximal part of the fistula. After embolization of coil, coronary angiography showed satisfactory result with the fistula's stump only (Figure 3). A day after the procedure the patient was discharged without any complication. The patient was free of symptoms during months of follow-up.

## 3. Discussion

Coronary artery fistulas have been recognized since 1965 [1]. The clinical presentation is dependent upon the severity of the shunt and may include symptoms of congestive heart failure, arrhythmias, and angina pectoris [2]. Current indications for coronary artery fistula closure are limited to patients with clinical symptoms or to asymptomatic patients with high-flow shunts to prevent the development of complications [3].

Therapy strategies of CAF are divided into conservative therapy and surgical and transcatheter coil embolization [4–6]. The success of surgery is high but has significant mortality and morbidity risk. Coil embolization is choice of treatment because of the cost effectiveness and less risk compared to surgery.

However, the treatment of a coronary artery fistula in a patient with single coronary artery anomaly is a challenge. Since it is a rare entity, there is no randomized clinical trial. This is the first case reporting a successful transcatheter coil embolization of a single coronary artery fistula. Embolization of a coronary fistula can be difficult, and complications may occur in patients with single coronary artery. However, if care has been taken to determine the precise anatomy of the single coronary artery to assure that the coil will not adversely affect normal coronary blood, embolization can be considered an alternative to surgery.
